# Increased Ammonium Toxicity in Response to Exogenous Glutamine in Metastatic Breast Cancer Cells

**DOI:** 10.3390/metabo12050469

**Published:** 2022-05-23

**Authors:** Violet A. Kiesel, Madeline P. Sheeley, Shawn S. Donkin, Michael K. Wendt, Stephen D. Hursting, Dorothy Teegarden

**Affiliations:** 1 Department of Nutrition Science, Purdue University, West Lafayette, IN 47907, USA; vkiesel@unc.edu (V.A.K.); msheeley@nd.edu (M.P.S.); 2 Department of Animal Science, Purdue University, West Lafayette, IN 47907, USA; shawn.donkin@oregonstate.edu; 3 Department of Medicinal Chemistry and Molecular Pharmacology, Purdue University, West Lafayette, IN 47907, USA; mwendt@purdue.edu; 4 The Purdue Center for Cancer Research, Purdue University, West Lafayette, IN 47907, USA; 5 Department of Nutrition, University of North Carolina at Chapel Hill, Chapel Hill, NC 27599, USA; hursting@email.unc.edu; 6 Lineberger Comprehensive Cancer Center, University of North Carolina at Chapel Hill, Chapel Hill, NC 27599, USA; 7 Nutrition Research Institute, University of North Carolina at Chapel Hill, Kannapolis, NC 28081, USA

**Keywords:** glutamine metabolism, metabolic reprogramming, ammonium toxicity, metastasis, breast cancer

## Abstract

Several cancers, including breast cancers, show dependence on glutamine metabolism. The purpose of the present study was to determine the mechanistic basis and impact of differential glutamine metabolism in nonmetastatic and metastatic murine mammary cancer cells. Universally labeled ^13^C_5_-glutamine metabolic tracing, qRT-PCR, measures of reductive–oxidative balance, and exogenous ammonium chloride treatment were used to assess glutamine reprogramming. Results show that 4 mM media concentration of glutamine, compared with 2 mM, reduced viability only in metastatic cells, and that this decrease in viability was accompanied by increased incorporation of glutamine-derived carbon into the tricarboxylic acid (TCA) cycle. While increased glutamine metabolism in metastatic cells occurred in tandem with a decrease in the reduced/oxidized glutathione ratio, treatment with the antioxidant molecule N-acetylcysteine did not rescue cell viability. However, the viability of metastatic cells was more sensitive to ammonium chloride treatment compared with nonmetastatic cells, suggesting a role of metabolic reprogramming in averting nitrogen cytotoxicity in nonmetastatic cells. Overall, these results demonstrate the ability of nonmetastatic cancer cells to reprogram glutamine metabolism and that this ability may be lost in metastatic cells.

## 1. Introduction

Glutamine is the second-most consumed nutrient in cancer cells, following glucose, and its metabolism is often required for cancer cell proliferation [1,2,3]. Glutamine has several potential cell fates, including incorporation into nascent peptides, contribution to nucleotide synthesis, participation in antiport exchange for other amino acids, including leucine, or catabolism [4,5,6,7,8]. In the catabolic pathway, glutamine is converted to glutamate and subsequently to α-ketoglutarate (αKG) for entry into the tricarboxylic acid (TCA) cycle for oxidation and energy production [9,10]. Previous literature has identified glutamine as a key metabolite for TCA cycle anaplerosis in cancer cells [11,12]. These various cell fates of glutamine highlight its potential roles in supporting the progression of cancer cells.

A battery of enzymes is required to mediate the conversion of glutamine to its metabolites in the catabolic pathway for entry into the TCA cycle. First, glutaminase (GLS) 1 and 2 convert glutamine to glutamate in a reaction that produces ammonium as a byproduct [13]. Conversion of glutamate to αKG is then mediated by one of three transaminases: glutamic oxaloacetic transaminase (GOT), glutamic pyruvic transaminase (GPT), and phosphoserine aminotransferase (PSAT), or glutamate dehydrogenase (GLUD1) [1,13]. Transaminase enzymes transfer the amine group from glutamine to an α-keto acid, producing an amino acid, while GLUD1 deaminates glutamate to produce ammonium. Of note, ammonium has previously been associated with decreased cell viability in models of cancer and untransformed cells, potentially through intracellular acidification and induction of apoptosis or through changes to N- and O-glycosylation of proteins [14,15,16].

In the current study, nonmetastatic M-Wnt and metastatic metM-Wnt^lung^ murine mammary cancer cell lines were employed to determine the mechanistic basis and impact of differential glutamine metabolism. M-Wnt cells were derived from spontaneously formed primary tumors in mouse mammary tumor virus (MMTV) Wnt-1 transgenic mice [17]. Following their isolation, M-Wnt cells were serially transplanted through five generations of severe-combined immunodeficient mice, and tumor cells were harvested from lung metastatic lesions to generate the metM-Wnt^lung^ cell line [17]. Implanting metM-Wnt^lung^ cells into the mammary fat pad results in mammary-to-lung metastasis in 50% of animals, whereas M-Wnt cells implanted into the fat pad do not form metastases in the liver or lung [17,18]. Previous work shows that metM-Wnt^lung^ cells have higher levels of oxidative metabolism compared with their nonmetastatic counterparts [17], suggesting that these cells may be prone to higher levels of nutrient oxidation, including glutamine oxidation. Therefore, this model was selected to determine the effect of glutamine concentration on glutamine reprogramming in different stages of cancer progression.

In the present studies, we hypothesized that metastatic cells lack the ability to adapt to increased glutamine metabolism in response to increasing glutamine concentration and that higher doses of glutamine reduce the viability of metastatic cells through the production of ammonium. These results suggest a metabolic vulnerability of metastatic compared to nonmetastatic cells and highlight targeting glutamine metabolism as a strategy to prevent metastatic progression.

## 2. Results

### 2.1. Exogenous Glutamine Concentration Reprograms Glutamine Metabolism in M-Wnt Cells

**Figure 1:**
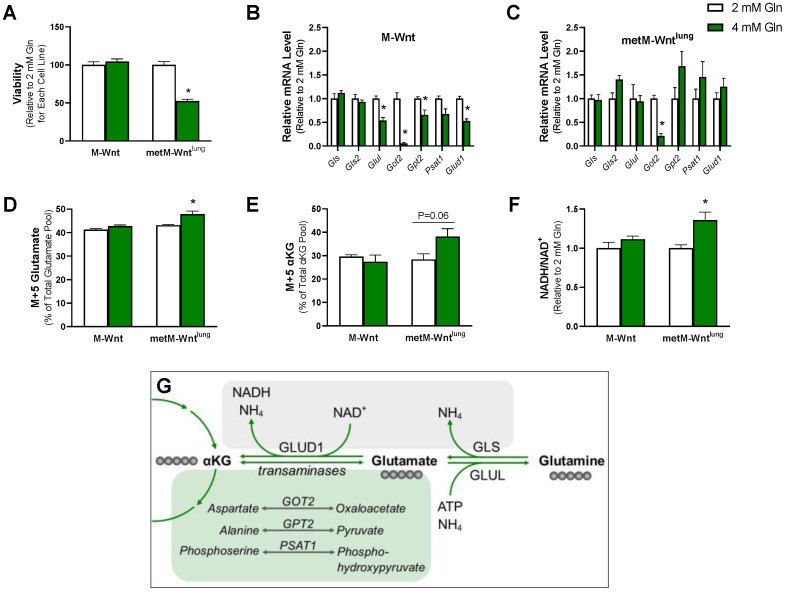
Effect of glutamine concentration on glutamine metabolism. (**A**) Viability of M-Wnt and metM-Wnt^lung^ cells maintained in 2 mM or 4 mM glutamine was assessed by MTT; (**B**,**C**) mRNA level of genes involved in glutamine metabolism in M-Wnt and metM-Wnt^lung^ cells was determined; (**D**,**E**) labeled ^13^C_5_-labeled glutamine was used to determine labeling of the downstream metabolites glutamate and α-ketoglutarate; (**F**) NADH/NAD^+^ ratios were measured. Overview of glutamine catabolism (**G**). Abbreviations: GLS—glutaminase; GLUL—glutamine synthetase; GLUD1—glutamate dehydrogenase; GOT2—glutamic oxaloacetic transaminase; GPT2—glutamic pyruvic transaminase; PSAT1—phosphoserine aminotransferase 1. The green box highlights transaminase reactions that are coupled with the conversion of glutamate to αKG; the grey box highlights ammonium-producing reactions. Grey circles indicate ^13^C labeled carbons in each metabolite if derived from exogenous universally labeled ^13^C glutamine. Results are expressed as means + SEM. Asterisk (*) indicates *p* < 0.05 relative to 2 mM glutamine.

M-Wnt and metM-Wnt^lung^ cells were maintained in 2 mM or 4 mM glutamine in order to determine whether variable glutamine concentrations affected the viability of nonmetastatic compared with metastatic cells. Higher glutamine conditions (4 mM) decreased the viability of metM-Wnt^lung^ cells by 48% (Figure 1A) but did not affect the viability of M-Wnt cells. In addition, we found that there was no difference in the viability of metM-Wnt^lung^ and M-Wnt cells when both cell lines were grown in 2 mM glutamine (data not shown). These results indicate that 4 mM glutamine decreases viability only in metastatic metM-Wnt^lung^ cells and suggest that nonmetastatic M-Wnt cells may regulate glutamine metabolism in order to maintain their viability.

In order to determine a mechanism by which M-Wnt cells, but not the metM-Wnt^lung^ cells, were able to adapt to 4 mM glutamine concentrations, expression of genes involved in glutamine metabolism was assessed in both glutamine concentrations. Compared with cells grown in 2 mM glutamine, culturing M-Wnt cells in 4 mM glutamine significantly decreased relative mRNA levels of *Glud1* by 47% and the transaminases *Got2* and *Gpt2* by 95% and 34%, respectively (Figure 1B), all of which are enzymes that catabolize glutamate to αKG [1]. In contrast, 4 mM glutamine suppressed the mRNA level of only *Got2* (79%) in metM-Wnt^lung^ cells (Figure 1C). There was a 48% decrease in glutamine synthetase (*Glul*) mRNA level, the enzyme which mediates the synthesis of glutamine from glutamate [1], in 4 mM compared with 2 mM glutamine culture conditions in M-Wnt cells (Figure 1B), whereas glutamine concentration did not affect *Glul* mRNA levels in metM-Wnt^lung^ cells (Figure 1C). These results indicate that glutamine concentration reduces the expression of genes related to glutamine catabolism in M-Wnt cells, and thus that increasing the glutamine concentration in these cells may inhibit glutamine catabolism.

To test the hypothesis that decreased expression of genes involved in glutamine catabolism functionally prohibits glutamine metabolism, metabolic tracing using universally labeled ^13^C_5_ glutamine was employed. Since glutamine contains five carbons and no carbon is lost in the conversion to glutamate or αKG, M+5 labeling of glutamate or αKG indicates that these metabolites were synthesized from labeled glutamine [19]. There was no enrichment of M+5 labeling of glutamate or αKG in M-Wnt cells in 4 mM compared to 2 mM glutamine (Figure 1D,E). In contrast, culturing metM-Wnt^lung^ cells in 4 mM glutamine significantly enriched the pool of M+5 glutamate (11%, Figure 1D) and trended towards the enrichment of M+5 αKG (34%, Figure 1E). These results collectively suggest that there is greater glutamine metabolism in metM-Wnt^lung^ cells when glutamine concentration is increased in the media to 4 mM compared with 2 mM concentrations. Conversely, increasing glutamine concentrations in M-Wnt cells modifies the expression of genes involved in glutamine catabolism, and pools of metabolites in the glutamine catabolic pathway are not enriched, suggesting downregulation of the glutamine catabolic pathway in response to increased exogenous glutamine concentrations.

### 2.2. Higher Glutamine Concentration Increases NADH/NAD^+^ Only in metM-Wnt^lung^ Cells

Metabolism of glutamine through the forward TCA cycle increases the production of NADH from NAD^+^, thus increasing the NADH/NAD^+^ ratio. Because the NADH/NAD^+^ ratio is an indicator of both the energetic and the reductive–oxidative (redox) status of the cell and is reported to be increased in breast cancer cells [20,21], the effect of glutamine concentration on the NADH/NAD^+^ ratio was assessed in both cell lines. Culturing cells in 4 mM glutamine significantly increased the NADH/NAD^+^ ratio by 36% in metM-Wnt^lung^ cells but had no effect on the NADH/NAD^+^ ratio in M-Wnt cells (Figure 1F). Collectively, these data suggest that increasing glutamine concentrations increases glutamine oxidation and NADH production in the TCA cycle in metM-Wnt^lung^ cells, whereas increasing glutamine concentration in M-Wnt cells does not increase the flow of carbon from glutamine into the TCA cycle or NADH production in M-Wnt cells.

### 2.3. Glutamine Levels Do Not Affect Oxidative Stress in M-Wnt Cells

**Figure 2:**
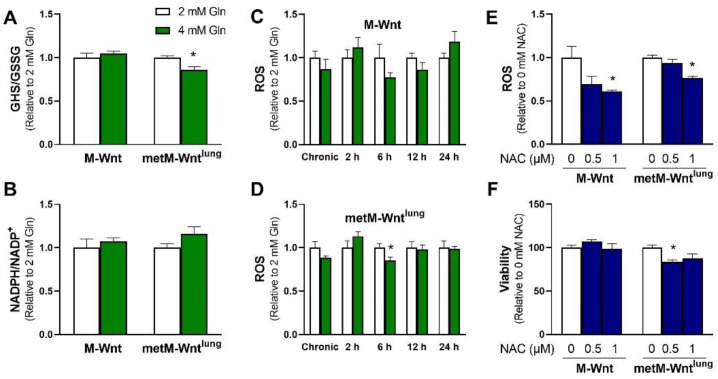
Effect of glutamine concentration on oxidative stress markers. (**A**) GSH/GSSG and (**B**) NADPH/NADP^+^ ratios were measured in M-Wnt and metM-Wnt^lung^ cells; (**C**,**D**) ROS levels were assessed in M-Wnt and metM-Wnt^lung^ cells chronically grown in 2 mM or 4 mM glutamine, and in cells grown in 4 mM glutamine for indicated times; (**E**,**F**) the effects of N-acetylcysteine (NAC) on ROS and viability were assessed in cells grown in 4 mM glutamine. Results are expressed as means + SEM. Asterisk (*) indicates *p* < 0.05 relative to 2 mM glutamine (in **A**,**D**) or relative to 0 mM NAC (**E**,**F**).

Because high levels of glutamine metabolism and NADH production in metM-Wnt^lung^ cells may increase metabolism-induced reactive oxygen species (ROS) generation and thus decrease cell viability, the impact of glutamine concentration on intracellular ROS and overall redox balance was determined. Two important redox systems used to neutralize intracellular ROS, the ratios of reduced/oxidized glutathione (GSH/GSSG) and NADPH/NADP^+^, were analyzed [22]. metM-Wnt^lung^ cells grown in 4 mM glutamine showed a 14% decrease in the GSH/GSSG ratio compared with 2 mM glutamine (Figure 2A), suggesting that 4 mM glutamine increases oxidative stress in metastatic cells. In contrast, the GSH/GSSG ratio was not affected by glutamine concentration in M-Wnt cells. However, 4 mM glutamine culture conditions did not affect the NADPH/NADP^+^ ratio in either cell line (Figure 2B). In order to determine if the decrease in GSH/GSSG in metM-Wnt^lung^ cells was associated with elevated oxidative stress, intracellular ROS levels were measured in cells grown in variable glutamine. The ROS levels in both M-Wnt and metM-Wnt^lung^ cells grown in 4 mM glutamine culture conditions were similar to their counterparts in 2 mM glutamine (Figure 2C,D). In order to determine if ROS levels transiently changed in response to increasing glutamine concentration, media was changed from 2 mM to 4 mM glutamine, and ROS levels were measured two, six, twelve, and twenty-four hours later. Short treatment with 4 mM glutamine did not increase ROS levels at any time point in either cell line (Figure 2C,D), suggesting that increased ROS and oxidative stress do not underlie the decrease in viability observed in metM-Wnt^lung^ cells cultured in 4 mM glutamine. Further, in order to determine if alleviating oxidative stress would rescue the viability of metM-Wnt^lung^ cells in 4 mM glutamine, cells were cultured with the antioxidant molecule N-acetylcysteine. While a 1 mM dose of N-acetylcysteine decreased ROS in M-Wnt (39%) and metM-Wnt^lung^ cells (24%, Figure 2E), this dose did rescue the viability of metM-Wnt^lung^ cells grown in 4 mM glutamine (Figure 2F). These data suggest that changes in redox balance in 4 mM glutamine do not affect cell viability of either nonmetastatic or metastatic cells, and an alternate mechanism underlies the decrease in viability of metM-Wnt^lung^ cells in 4 mM glutamine.

### 2.4. Ammonium Reduces Viability of metM-Wnt^lung^ Cells

**Figure 3:**
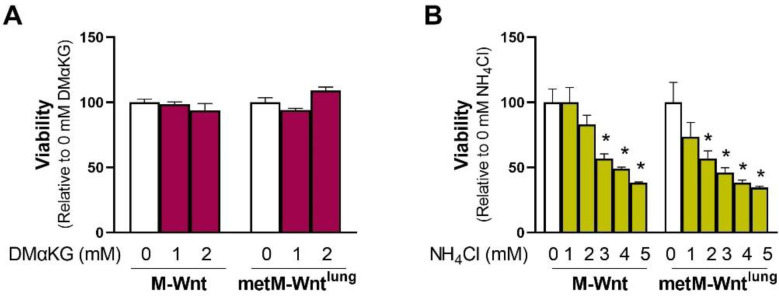
Effect of dimethyl α-ketoglutarate and ammonium chloride on cell viability. (**A**) The effect of exogenous addition of a membrane-permeable form of α-ketoglutarate (dimethyl α-ketoglutarate, DMαKG) or (**B**) ammonium chloride (NH_4_Cl) on viability was assessed in cells constitutively grown in 2 mM glutamine. Results are expressed as means + SEM. Asterisk (*) indicates *p* < 0.05 relative to 0 mM ammonium chloride (**B**).

A potential mechanism by which 4 mM glutamine decreases the viability of metM-Wnt^lung^ cells may be a result of increasing concentrations of carbon in the TCA cycle or nitrogen in the cell. In order to test the former mechanism related to the supply of carbon, cells grown in 2 mM glutamine were treated with 1 or 2 mM dimethyl α-ketoglutarate (DMαKG), a membrane-permeable form of αKG. Neither dose of DMαKG affected viability in either cell line (Figure 3A), suggesting that the carbon supplied in 4 mM glutamine culture conditions is not the source of decreased viability in metM-Wnt^lung^ cells. During glutaminolysis, the amide nitrogen of glutamine is lost as ammonium in the glutaminase reaction, and the amine nitrogen is either lost as ammonium in the GLUD1 reaction or transferred to an α-keto acid by transaminases [1,10]. Therefore, cells growing in 2 mM glutamine were treated with ammonium chloride to determine if increasing ammonium concentrations reduces the viability of metM-Wnt^lung^ cells. While 2 mM ammonium chloride significantly decreased the viability of metM-Wnt^lung^ cells by 43%, the same dose had no effect on the viability of M-Wnt cells (Figure 3B). Similarly, 3 mM ammonium chloride suppressed viability by 54% in metM-Wnt^lung^ cells and 43% in M-Wnt cells (Figure 3B), indicating that the viability of metM-Wnt^lung^ cells is more sensitive to ammonium chloride treatment compared with M-Wnt cells. In agreement with this, the lethal dose 50 (LD_50_) of ammonium chloride was 2.0 mM in metM-Wnt^lung^ cells and 3.1 mM in M-Wnt cells. These data indicate that metM-Wnt^lung^ cells are sensitive to ammonium toxicity and suggest that the production of ammonium as a byproduct of glutamine catabolism may underly their decreased viability at higher glutamine concentrations.

### 2.5. Glutamine Concentration Does Not Upregulate Ammonium Detoxification Genes

**Figure 4:**
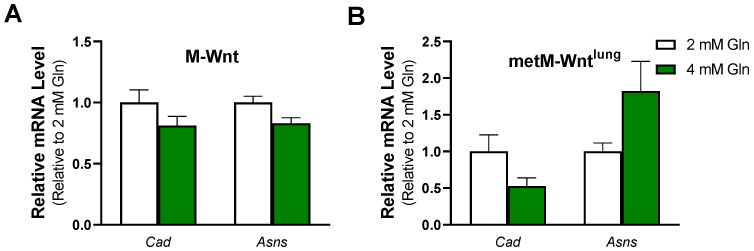
Effect of glutamine concentration on ammonium detoxification genes. Relative mRNA level of carbamoyl-phosphate synthetase 2 (Cad) and asparagine synthetase (Asns) was determined by qRT-PCR in (**A**) M-Wnt and (**B**) metM-Wnt^lung^ cells grown in 2 or 4 mM glutamine.

There are several cellular mechanisms to detoxify ammonium which may contribute to improved survival of M-Wnt cells in 4 mM glutamine. For instance, ammonium is condensed with αKG to form glutamate through the activity of GLUD1 or with glutamate to form glutamine through the activity of GLUL [23]. In M-Wnt cells, *Glud1* mRNA levels were decreased by 47%, and *Glul* mRNA levels were decreased by 46% in 4 mM glutamine compared with 2 mM glutamine (Figure 1B), suggesting that these genes do not contribute to ammonium detoxification in M-Wnt cells. In contrast, neither gene was regulated by glutamine concentration in metM-Wnt^lung^ cells (Figure 1C). A third cellular ammonium detoxification strategy is utilizing ammonium for pyrimidine synthesis via carbamoyl-phosphate synthetase 2 (CAD) [23]. Glutamine concentration did not affect *Cad* mRNA levels in either cell line tested (Figure 4A,B). A final mechanism to decrease ammonium production involves the upregulation of asparagine synthetase (ASNS). In the ASNS reaction, the amide nitrogen from glutamine is transferred to aspartate for asparagine synthesis rather than being lost as ammonium [23]. Similar to *Cad*, there was no effect of glutamine concentration on mRNA levels of *Asns* in either cell line (Figure 4A,B). These data suggest that neither cell line upregulates genes involved in ammonium detoxification in response to 4 mM glutamine concentrations and may suggest that M-Wnt cells rely on suppression of glutamine catabolism to avoid ammonium toxicity and maintain viability in elevated glutamine concentrations.

### 2.6. Ammonium Does Not Modify Gene Expression in M-Wnt Cells

**Figure 5:**
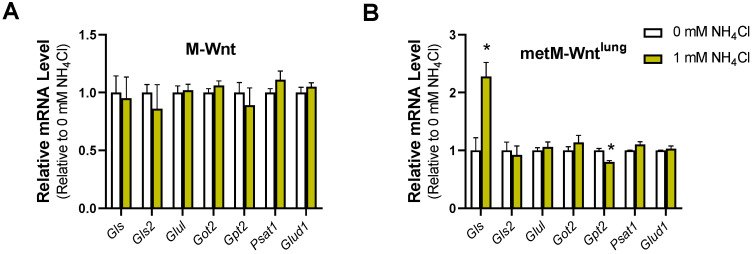
Effect of ammonium chloride treatment on mRNA abundance. Relative mRNA level was determined by qRT-PCR in (**A**) M-Wnt and (**B**) metM-Wnt^lung^ cells grown in 2 mM glutamine with 1 mM ammonium chloride (NH_4_Cl) or sodium chloride (Ctrl) for 48 h. Results are expressed as means + SEM. Asterisk (*) *p* < 0.05 relative to Ctrl.

Finally, we aimed to determine whether ammonium chloride treatment recapitulates the changes in mRNA levels observed in M-Wnt cells cultured in 4 mM glutamine compared to 2 mM glutamine. Ammonium chloride treatment had no effect on the expression of genes related to glutamine catabolism in M-Wnt cells (Figure 5A). Ammonium chloride treatment increased *Gls* expression and decreased *Gpt2* expression in metM-Wnt^lung^ cells (Figure 5B). These results indicate that increased ammonium concentrations are not responsible for the changes in gene expression induced by 4 mM glutamine in M-Wnt cells and suggest that another mechanism by which gene expression is regulated remains to be identified in M-Wnt cells.

## 3. Discussion

Glutamine is a key nutrient that is often required to support proliferation and redox balance in proliferating cancer cells [1]. In this work, we investigated the effects of variable glutamine concentrations on metabolic reprogramming and viability in metastatic compared to nonmetastatic murine mammary cancer cells. Metastatic metM-Wnt^lung^ cells displayed increased conversion of glutamine to glutamate in response to increasing glutamine concentration, whereas nonmetastatic M-Wnt cells had no change in glutamine metabolism in 4 mM glutamine, suggesting that models of breast cancer at different stages of progression utilize glutamine differently.

Previous work demonstrates a high level of variability in glutamine utilization between different types of cancer, and limited research has shown that glutamine utilization varies with the degree of cancer progression [1]. For example, previous literature shows that more aggressive prostate cancer cell lines increase glutamine flux into the TCA cycle compared with their less metastatic counterparts [24]. In agreement with this, the present work shows that metastatic metM-Wnt^lung^ cells have increased glutamine catabolism in response to increasing glutamine concentrations, whereas M-Wnt cells reprogram glutamine metabolism in response to 4 mM glutamine (Figure 1).

While glutamine metabolism and dependence vary both within and between types of cancer, previous literature has identified a unique effect of glutamine within the lung microenvironment. Specifically, models of primary lung cancer or breast cancer cells that metastasized to the lung show decreased utilization of glutamine compared with surrounding normal tissue, suggesting that glutamine is either dispensable or limiting for the survival of cancer cells at the lung [25,26]. In accordance with this, the present work may suggest that high levels of glutamine metabolism limit the survival of lung-tropic cancer cells. While evidence suggests that metastatic cells have enhanced overall metabolic plasticity [27], the data presented here are consistent with evidence suggesting that cancer cells increase utilization of specific metabolic pathways or substrates based on their microenvironment [28,29,30,31,32].

Variability in glutamine metabolism may be partially explained by differences in driver mutations that occur across different types of cancer. For example, glutaminase expression is partially controlled by oncogene expression, including expression of c-Myc and N-Myc, K-ras, and phosphatidylinositol-4,5-bisphosphate 3-kinase catalytic subunit alpha (PIK3CA) [33,34,35,36,37]. These data suggest that more metastatic cancer cells, which have accrued more genetic mutations, may have increased upregulation of glutaminase and glutamine metabolism. This observation is in line with our current results, as there was little change in the expression of genes involved in glutamine metabolism in metM-Wnt^lung^ cells cultured in 4 mM glutamine. In addition, increasing glutamine concentrations to 4 mM significantly enriched M+5 glutamate from labeled glutamine in metM-Wnt^lung^ cells (Figure 1). These data may suggest that enzymes involved in glutamine catabolism, including glutaminase, are constitutively activated in metM-Wnt^lung^ cells independent of glutamine concentration. In addition, stimulation of cultured ST2 murine bone marrow stromal cells with Wnt3a increases GLS protein expression and activity [38]. Stimulation of GLS through Wnt signaling is proposed to be through both direct effects of Wnt pathway activation, as well as indirectly through the transcription of c-Myc [38,39]. For example, in colon cancer cells, enhanced β-catenin signaling increased c-Myc and glutamine metabolism [39]. These data collectively emphasize the importance of not only total mutation accrual but also specifically the dysregulation of Wnt signaling for the regulation of glutamine metabolism. Thus, these data highlight the relevance of utilizing an MMTV-Wnt-1-driven cell model of breast cancer progression in the present study.

A limitation of the current study is that the specific mechanism underlying increased glutamine catabolism in metM-Wnt^lung^ cells has not been identified. Given the array of enzymes that mediate the conversion of glutamine to glutamate and subsequently glutamate to αKG, analysis of enzyme activity along this catabolic pathway may provide useful information in determining which specific enzymes contribute to the metabolic shift observed in metM-Wnt^lung^ cells. However, it is important to recognize that the mechanisms driving the response to low levels of glutamine (‘addiction’) are likely not the same as those driving the response to high compared with moderate glutamine levels [11,40], limiting the use of inhibitors of enzymes in the glutaminolysis pathway to define the mechanisms of the response to high glutamine noted in the metastatic cells described in the current study. In addition to analyzing enzyme activity, the work presented in this manuscript warrants further investigation of glutamine metabolism in additional pairs of metastatic and nonmetastatic breast cancer cells with different oncogenic drivers, as different driver mutations are expected to have variable effects on glutamine flux. Continuous efforts to expand what is known in this field will be useful in establishing relationships between glutamine metabolism and mutation profiles in a variety of cancers, which may aid in the elucidation of which tumors respond best to treatments targeting glutamine metabolism.

The mechanism by which M-Wnt cells detect and respond to higher levels of glutamine requires further investigation. One potential mode of signal transduction that translates metabolic stress information into changes in transcriptional activity is oxidative stress. Intermediate levels of ROS, which are produced secondary to glutamine oxidation and ATP production at the electron transport chain, can stimulate transcription [41]. However, increasing glutamine concentration had no effect on measures of ROS or redox balance in M-Wnt cells (Figure 2), suggesting that increasing glutamine concentration does not increase oxidative stress in these cells. Another potential mechanism to regulate the expression of genes related to glutamine metabolism is through ammonium. However, our results demonstrate that treatment with exogenous ammonium chloride did not recapitulate the changes in gene expression observed in 2 mM compared with 4 mM glutamine. As such, the mechanism driving changes in gene expression in M-Wnt cells in higher glutamine concentrations has not been identified.

A potential mechanism underlying decreased viability in metM-Wnt^lung^ cells in 4 mM glutamine involves ammonium production. Previous work has shown that ammonium is produced primarily in the glutaminase reaction and decreases cell viability [13]. Our results show that metM-Wnt^lung^ cells have increased metabolism of glutamine to glutamate in higher glutamine concentrations (Figure 1), which may suggest that increased ammonium production secondary to glutaminolysis underlies the decreased viability of metM-Wnt^lung^ cells. Indeed, exogenous ammonium chloride treatment more dramatically decreased the viability of metM-Wnt^lung^ cells compared with M-Wnt cells, potentially explaining their reduced viability in 4 mM glutamine (Figure 3). Interestingly, M-Wnt cells were relatively resistant to ammonium chloride treatment, suggesting nonmetastatic cells possess cellular mechanisms for ammonium detoxification.

Further research is required to determine how M-Wnt cells mediate their resistance to exogenous ammonium chloride treatment. Several cellular strategies to increase ammonium assimilation or limit ammonium production have been identified in cancer cells, thereby averting ammonium toxicity. For example, breast cancer cells were shown to assimilate ammonium through GLUD1 activity by increasing the conversion of αKG to glutamate [42]. In addition, cancer cells cultured in hypoxia shunt amide nitrogen from glutamine into pyrimidine synthesis through upregulation of CAD [43]. Finally, cells may utilize ASNS to transfer the amide nitrogen of glutamine to aspartate [23], thus limiting ammonium production at the GLS reaction by competing for substrate availability. In the present study, mRNA levels of genes involved in ammonium assimilation or the rerouting of the amide nitrogen from glutamine were not upregulated in 4 mM glutamine in either cell line (Figure 4). As such, it is currently unclear how M-Wnt cells mediate their resistance to exogenous ammonium chloride treatment and how this mechanism may be reduced in metM-Wnt^lung^ cells.

The data presented in this study support that cancer cells at different stages of progression differentially reprogram metabolism in response to glutamine availability. While nonmetastatic cells appear to utilize a system to detect and respond to high levels of glutamine to maintain their viability, this ability is not present in metastatic cells. Further research is needed to elucidate the differential mechanisms by which higher levels of glutamine are translated into changes in transcriptional activity and in resistance to ammonium chloride exposure between M-Wnt cells versus metM-Wnt^lung^ cells. Overall, the current results provide new evidence of differences in metabolic reprogramming of glutamine that occur over the course of cancer progression and may highlight a metabolic vulnerability of metastatic breast cancer cells.

## 4. Materials and Methods

### 4.1. Chemicals and Reagents

N-acetylcysteine, ammonium chloride, and dimethyl α-ketoglutarate were purchased from Sigma (St. Louis, MO, USA).

### 4.2. Cell Culture

M-Wnt and metM-Wnt^lung^ cells are models of nonmetastatic and metastatic murine mammary cancer, respectively [17]. Both cell lines were constitutively cultured in DMEM (Sigma) with 5 mM glucose and either 2 mM or 4 mM glutamine. These two concentrations of glutamine were selected for analysis as they reflect typical levels of glutamine found in both RPMI and DMEM cell culture media and thus have broad applicability in cell culture. Complete cell culture media contained a final concentration of 1% penicillin/streptomycin antibiotic solution (Gibco, Waltham, MA, USA) and 10% fetal bovine serum (Gibco).

### 4.3. MTT Assay

Cells were seeded at equal densities into 96-well plates, attached overnight, and treated with indicated reagents for 48 h. Cell viability was determined through a 3-(4,5-dimethylthiazol-2-yl)-2,5-diphenyltetrazolium bromide (MTT) assay according to the manufacturer’s recommendations (Sigma, St. Louis, MO, USA). Briefly, cell culture media was replaced with 1X MTT reagent in serum-free media, and cells were incubated for two hours at 37 °C. Following incubation, media was removed, and crystals were dissolved in dimethyl sulfoxide (DMSO). Absorbance was measured at 570 nm.

### 4.4. RNA Isolation and qRT-PCR

RNA was isolated from cell samples using TRI-Reagent (Molecular Research Center, Cincinnati, OH, USA) following the manufacturer’s protocol. RNA was reverse-transcribed to cDNA with MMLV reverse transcriptase (Promega, Madison, WI, USA). qRT-PCR was conducted with a LightCycler 480 instrument with LightCycler 480 SYBR Green I Master Mix (Roche, Indianapolis, IN, USA) using primers listed in Table 1. The comparative Ct method (2^−ΔCt^) was used for data normalization, with results corrected for data from 2 mM glutamine culture conditions.

### 4.5. Glutamine Metabolic Tracing

Cells constitutively grown in 2 mM or 4 mM glutamine were grown to 80% confluence. Media was removed and replaced with fresh media containing 100% of either 2 mM or 4 mM universally labeled ^13^C_5_-glutamine for two hours at 37 °C prior to harvesting samples in 70% ethanol heated to 70 °C. As an internal standard, norvaline (1 µg norvaline/mL sample) was added to each sample, vortexed, and incubated at 95 °C for 5 min. Samples were cooled on ice for 5 min and centrifuged at 18,000× *g* for 5 min at room temperature. Cell pellets were analyzed for protein content with a bicinchoninic acid (BCA) assay (ThermoFisher, Waltham, MA, USA). Supernatants were dried and derivatized with methoxylamine hydrochloride in pyridine and prepared with N-tert-butyldimethylsilyl-N-methyltrifluoroacetamide with 1% (wt/wt) tert-butyldimethylchlorosilane for analysis with gas chromatography-mass spectrometry (Thermo TSQ 8000 triple quadrupole mass spectrometer coupled with a Thermo Trace 1310 gas chromatography) [44].

### 4.6. NAD^+^/NADH Assay

Cells were seeded into the white-walled clear bottom 96-well plates (Corning, Corning, NY, USA). The next day, cells were washed once with 1X calcium and magnesium-free phosphate-buffered saline (PBS), and NAD^+^ and NADH were detected using NAD^+^/NADH-Glo Assay kit (Promega) according to the manufacturer’s instructions. Luminescence was measured using a Synergy H1 Multi-Mode reader.

### 4.7. Oxidative Stress Assays

Cells were seeded into the white-walled clear bottom 96-well plates (Corning). On day two, ratios of NADPH/NADP^+^ and GSH/GSSG were measured with NADP^+^/NADPH-Glo and GSH/GSSG-Glo Assays (Promega) according to the manufacturer’s instructions. Luminescence was measured using a Synergy H1 Multi-Mode reader.

### 4.8. ROS Assay

Cellular reactive oxygen species (ROS) levels were measured using 2′,7′-dichlorofluorescin diacetate (DCFH-DA) [45,46]. Cells were plated into black-walled clear bottom 96-well plates (Corning). For time-course ROS assays, cells grown in 2 mM glutamine were seeded, and the next day, the media was changed to 4 mM glutamine for two, six, twelve, or twenty-four hours. Following treatment, media was removed, and cells were washed once with PBS. Cells were incubated in the dark at 37 °C in 10 μM DCFH-DA in PBS for 20 min. Fluorescence was measured using a Synergy H1 Multi-Mode reader (excitation/emission 485/530 nm). Fluorescence measures were normalized to cell viability, as measured by MTT.

### 4.9. Statistical Analysis

Values are presented as means + SEM. Statistics were analyzed using SAS software version 9.4, and *p* values < 0.05 were considered significant.

## Figures and Tables

**Table 1:** Primers used for qRT-PCR.

Gene	Primer
*Asns*	Forward: 5′- CACAAGGCGCTACAGCAAC-3′ Reverse: 5′- CCAGCATACAGATGGTTTTCTCG-3′
*Cad*	Forward: 5′- GGGGAAGTGGTGTTTCAGACC-3′ Reverse: 5′- CGTAGTTGCCGATGAGAGGAT-3′
*Glud1*	Forward: 5′-CCCAACTTCTTCAAGATGGTGG-3′ Reverse: 5′-AGAGGCTCAACACATGGTTGC-3′
*Glul1*	Forward: 5′-TGAACAAAGGCATCAAGCAAATG-3′ Reverse: 5′-TGAACAAAGGCATCAAGCAAATG-3′
*Gls*	Forward: 5′-CTACAGGATTGCGAACATCTGAT-3′ Reverse: 5′-ACACCATCTGACGTTGTCTGA-3′
*Gls2*	Forward: 5′-CAGAGGGACAGGAGCGTATC-3′ Reverse: 5′-TTCTTTCGGAATGCCTGAGTC-3′
*Got2*	Forward: 5′-GGACCTCCAGATCCCATCCT-3′ Reverse: 5′-GGTTTTCCGTTATCATCCCGGTA-3′
*Gpt2*	Forward: 5′-AACCATTCACTGAGGTAATCCGA -3′ Reverse: 5′-GGGCTGTTTAGTAGGTTTGGGTA -3′
*Psat1*	Forward: 5′-CAGTGGAGCGCCAGAATAGAA-3′ Reverse: 5′-CCTGTGCCCCTTCAAGGAG-3′
*18S*	Forward: 5′-ATCCCTGAGAAGTTCCAGCA-3′ Reverse: 5′-CCTCTTGGTGAGGTCGATGT-3′

## Data Availability

Not applicable.
